# Effects of *Staphylococcus aureus* Bacteriophage K on Expression of Cytokines and Activation Markers by Human Dendritic Cells In Vitro

**DOI:** 10.3390/v10110617

**Published:** 2018-11-08

**Authors:** Helen R. Freyberger, Yunxiu He, Amanda L. Roth, Mikeljon P. Nikolich, Andrey A. Filippov

**Affiliations:** Department of Bacteriophage Therapeutics, Bacterial Diseases Branch, Walter Reed Army Institute of Research, Silver Spring, MD 20910, USA; helen.r.freyberger.ctr@mail.mil (H.R.F.); yunxiu.he.ctr@mail.mil (Y.H.); amanda.l.roth.mil@mail.mil (A.L.R); mikeljon.p.nikolich.civ@mail.mil (M.P.N.)

**Keywords:** *Staphylococcus aureus*, bacteriophage K, human dendritic cells, cytokines and activation markers

## Abstract

A potential concern with bacteriophage (phage) therapeutics is a host-versus-phage response in which the immune system may neutralize or destroy phage particles and thus impair therapeutic efficacy, or a strong inflammatory response to repeated phage exposure might endanger the patient. Current literature is discrepant with regard to the nature and magnitude of innate and adaptive immune response to phages. The purpose of this work was to study the potential effects of *Staphylococcus aureus* phage K on the activation of human monocyte-derived dendritic cells. Since phage K acquired from ATCC was isolated around 90 years ago, we first tested its activity against a panel of 36 diverse *S. aureus* clinical isolates from military patients and found that it was lytic against 30/36 (83%) of strains. Human monocyte-derived dendritic cells were used to test for an in vitro phage-specific inflammatory response. Repeated experiments demonstrated that phage K had little impact on the expression of pro- and anti-inflammatory cytokines, or on MHC-I/II and CD80/CD86 protein expression. Given that dendritic cells are potent antigen-presenting cells and messengers between the innate and the adaptive immune systems, our results suggest that phage K does not independently affect cellular immunity or has a very limited impact on it.

## 1. Introduction

*Staphylococcus aureus* is a leading cause of human bacteremia, infective endocarditis, osteoarticular, skin and soft tissue, pleuropulmonary, and device-related infections [1]. Clinical isolates of *S. aureus* demonstrate frequent multidrug resistance, the most important of which is methicillin and vancomycin resistance [2]. Formation of mature biofilms increases *S. aureus* persistence in tissues and medical implants and causes additional antibiotic tolerance [3]. The prevalence of multidrug-resistant *S. aureus* infections leaves physicians with limited options for antibacterial therapy and demands new therapies with current interest focused on bacteriophages (phages). Phages have shown efficacy against experimental *S. aureus* infections using several animal models, as well as promising results in humans [4].

There is a reasonable concern about the immunogenicity of phages and whether it could impact their in vivo efficacy [5,6,7]. Conflicting data on phage immunomodulatory properties, including their effects on inflammation, interactions with phagocytes, T and B cells, the fate of phage inside phagocytes, phage intracellular activity, cytokine stimulation, induction of antibody production, phage-neutralizing effects of the antibodies, and the impacts of phage immunogenicity on their therapeutic efficacy have made it difficult to interpret and draw overarching conclusions about the true impact of phage therapy (for reviews, see References [8,9,10]). For example, a recent publication described a marked stimulation of pro-inflammatory cytokines released by human PBMC and immortalized cell lines HT-29 and Caco-2 following exposure to four different *Escherichia coli* phages [11]. In fact, the levels of cytokines IL-6 and TNFα in phage-treated samples were comparable to those observed in the samples treated with lipopolysaccharide (LPS). These unusual results raised the question about whether possible insufficient purification of the phage preparations and LPS contamination compromised the interpretation of the data [12].

In contrasting studies, there was a complete lack of cytokine response to phage treatment. For example, enterobacteriophage T4 or its head proteins had no effect on the production of inflammatory mediators and inflammation-related factors including IL-1α, IL-1β, IL-2, IL-6, IL-10, IL-12 p40/p70, IFN-γ, TNFα, MCP-1, MIG, etc. using both human blood cells in vitro and an in vivo mouse model [13]. After oral administration of three *E. coli* O157:H7 phages to mice, all cytokine levels remained within normal ranges [14]. Purified phages T4 and A3/R had no effect on differentiation of human dendritic cells while crude phage lysates significantly reduced the expression of markers associated with differentiation [15]. Interestingly, mouse dendritic cells exposed to phage T4 had enhanced anti-tumor effect [16].

Several publications indicate that phages may have potent anti-inflammatory effects. For example, phages reduced cellular infiltration of mouse skin transplants and the levels of pro-inflammatory factor NF-κB [7]. Phage therapy in humans resulted in a dramatic reduction of C reactive protein values and leukocyte counts, which was unparalleled by a reduction in live bacteria counts [17]. Phage T4 tail protein gp12 identified as a receptor-binding protein was demonstrated to bind LPS and reduce LPS-induced inflammation in mice [18]. Another team observed the reduction of *S. aureus*-mediated expression of inflammatory cytokines in mice caused by phage SLPW [19].

Current literature provides evidence that even phages that target the same bacterial pathogen may yield different effects. Shiley et al. [20] have recently used a human lung in vitro model and observed a phage PEV2-mediated increase in IL-6 and TNFα production, yet another *Pseudomonas aeruginosa* phage, DMS3, did not change the levels of these cytokines. It was found that even the same phage may or may not induce inflammatory response depending on the approach used. Commercially available *S. aureus* phage SATA-8505 did not induce inflammatory responses in peripheral blood mononuclear cells (PBMC) but did induce IFN-γ production in primary keratinocyte cultures and elicited inflammatory responses in mice [21].

It was demonstrated that some phages can be ingested by phagocytes [22,23] but there are conflicting data on phage influence on phagocytosis of bacteria and intracellular bacterial lysis by phage. Phage therapy against different infections did not affect the ability of patients’ peripheral blood monocytes and polymorphonuclear neutrophils to kill *E. coli*, *Enterococcus faecalis*, *P. aeruginosa* or *S. aureus* [24]. Acceleration of turnover of circulating neutrophils but a decrease in their bactericidal activity was observed in patients who received phage against suppurative *S. aureus* infections [25]. In another study, increased phagocytosis by neutrophils was noted in patients with successful results of phage therapy [8]. In a recent work, Roach et al. [26] observed a neutrophil-phage synergy that was important for resolution of *P. aeruginosa* pneumonia in mice treated with phage PAK_P1: Mice depleted of their neutrophils were completely unresponsive to inhaled phage treatment.

Some phages are capable of penetrating phagocytes to kill internalized bacteria. An *S. aureus* phage MSa could penetrate into murine macrophages (but only using *S. aureus* cells as a carrier) and destroy the bacteria inside macrophages [27]. On the contrary, bacteriophage TM4 did not show the ability to be phagocytosed by macrophages and to kill intracellular *Mycobacterium tuberculosis* and *Mycobacterium avium*, though another phage, D29, was able to enter macrophages and kill the mycobacteria inside them [28]. Our previous data suggest that phage φA1122 is not able to get inside mouse macrophages either alone or using *Yersinia pestis* cells as a carrier and thus cannot kill the bacteria inside macrophages [29].

Investigative work on the relationship between the adaptive immune response and phages is also inconsistent. In some cases, phage-neutralizing antibodies were found in patient sera even before phage administration, suggesting pre-exposure to a common phage antigen or epitope found in the environment [8]. However, the titers of preexisting antibodies were as low as 1:10–1:100, while the local administration of phage resulted in much higher titers, up to 1:1500 [8]. Surprisingly, not only antibodies to T4 tail proteins that occlude the receptor-binding part of the phage caused phage inactivation, but also anti-head immunization reduced phage activity [30]. Interestingly, no antibody response was observed in volunteers who received T4 phage orally [31] and very low titers of phage-neutralizing antibodies were found in patients after multiple oral administrations of a therapeutic phage [8,32]. Anti-phage activity of sera of patients that received phage therapy also depended on phage type and did not impact the therapeutic outcome [32,33,34].

Thus multiple publications have shown different innate and adaptive immunity responses to phages, which requires additional studies on their immunomodulatory properties. The purpose of this work was to investigate effects of *S. aureus* phage K on activation of human monocyte-derived dendritic cells. Two experiments using different batches of human CD14+ monocytes showed that the K phage had very limited impact on the expression of pro- and anti-inflammatory cytokines, or activation factors. Since dendritic cells capture, process and present antigens to T cells and thus link the innate and adaptive immune systems, our data may indicate that *S. aureus* phage K has limited to no effect on cellular immunity.

## 2. Materials and Methods

### 2.1. Bacterial Strains and Media

*S. aureus* strains used in this work are described in Table 1. The cultures were grown in liquid Heart Infusion Broth (HIB) or on plates of 1.5% HIB agar (BD: Becton, Dickinson and Co., Franklin Lakes, NJ, USA). For phage plating, 0.7% semisolid HIB agar was overlaid on 1.5% HIB agar.

### 2.2. Bacteriophage Propagation and Purification

Phage K was purchased from the American Type Culture Collection (ATCC, Manassas, VA, USA). The phage was propagated on *S. aureus* NSCO308 (Table 1). Four milliliters of overnight bacterial culture was added to 400 mL of HIB in a 2-L flask and incubated at 37 °C with shaking at 200 rpm until the optical density at 600 nm (OD600) became 0.1–0.2 (ca. 10^8^ CFU/mL) that corresponds mid-log phase of the culture growth. The phage was added at a multiplicity of infection (MOI) of 0.1, the culture was thoroughly but carefully mixed, and phage was allowed to adsorb for 15 min at 37 °C without shaking. The mix was incubated at 37 °C and 100 rpm for at least 4 h with periodical measuring of OD600 until it dropped to 0.05 or less. Sodium chloride powder was added to 1 M, gently solved on a rocking platform; the lysate was incubated in an ice bath for one hour and centrifuged for 10 min at 4 °C and 11,000× *g* to remove the bacterial debris. PEG 8000 was added to the supernatant up to 10% (*w*/*v*), dissolved on a rocking platform and the mix was incubated in an ice bath for one hour. Phage particles were precipitated by centrifugation for 10 min at 4 °C and 11,000× *g* in two separate tubes and the supernatant was removed. The pellets were dried for 5 min at room temperature; one of them was resuspended in 3 mL of SM buffer (Teknova, Hollister, CA, USA), filter sterilized using a sterile 0.22-µm membrane, aliquoted in freeze vials, wrapped with aluminum foil and stored at 4 °C (phage stock for long storage). Another pellet was resuspended in phosphate-buffered saline (PBS, Gibco, Gaithersburg, MD, USA) and split into two parts, both for studies with human dendritic cells. One part was filter sterilized and stored at 4 °C. The second part of phage suspension in PBS was placed in Spectra/Por DispoDialyzers (MWCO = 15,000, Repligen Corporation, Waltham, MA, USA), dialyzed overnight at 4 °C against 1 L of PBS, then filter sterilized and stored at 4 °C.

### 2.3. Phage Efficiency of Plating (EOP) Tests

Five-milliliter aliquots of melted 0.7% HIB agar were cooled down to 47 °C, mixed with 0.1 mL of overnight culture of each tested *S. aureus* strain, overlaid on 1.5% HIB agar plates and left to solidify for 5 min at room temperature with half-open lids. Serial 10-fold dilutions of the phage K SM buffer stock from 10^−1^ to 10^−8^ were prepared in SM buffer using a sterile 96-well flat-bottom plate, and 2 µL of each dilution were spotted on the top agar with an 8-channel pipette. The drops were allowed to dry, the plates were flipped and incubated overnight at 37 °C. Plaques were counted and phage titers were calculated for each strain. The titer obtained on the propagation strain (NSCO308) was taken for the efficiency of plating = 1 and EOPs for the rest of the strains were calculated by dividing phage K titers on them into the titer on NSCO308. *S. aureus* culture was considered as phage-susceptible when the phage was able to form isolated plaques from the highest dilutions (i.e., productive phage infection was observed), and the absence of plaquing was considered as phage resistance.

### 2.4. Monocyte-Derived Dendritic Cells

Primary human CD14+ monocytes were purchased from ATCC. Dendritic Cell Differentiation Kits (R&D Systems, Minneapolis, MN, USA) were used according to kit directions to convert the monocytes to immature monocyte-derived dendritic cells. Briefly, cells were plated in 24-well plates at 10^6^ cells/mL in “differentiation medium” from the kit containing IL-4 and GM-CSF with half-volume medium changes on days 3, 5 and 7. On day 7, cells were treated with either positive control “activation medium” containing tumor necrosis factor (TNF), phage-treated medium containing phage K at a final concentration of 10^8^ PFU/mL (100 PFU/cell), or negative control medium with a volume of PBS equal to that of the phage preparation. At 24 h intervals after treatment, triplicate sample wells were collected by pipetting off medium into a separate tube, briefly centrifuging to remove cellular debris, and freezing aliquots at −80 °C for later analysis. Cells were rinsed with PBS, resuspended with TrypLE Express (Gibco), rinsed again with PBS, and fixed in 4% formalin in PBS for 10 min in a shaking incubator at 37 °C. Cells were rinsed twice with PBS, resuspended in PBS and stored at 4 °C until analysis. Dendritic cell phenotype was confirmed by staining for CD11c and CD209 during analysis.

### 2.5. Flow Cytometry

Formalin-fixed cell samples were suspended in staining buffer (10% fetal bovine serum in PBS). Samples were treated with Fc block, then stained with either staining buffer including APC-labeled anti-CD209 (BD), BV421-labeled anti-CD80 (BD), PerCP-Cy5.5-labeled anti-CD11c (BD), PE-Cy7-labeled anti-CD86 (BD), FITC labeled anti-MHC I HLA-ABC (Invitrogen, Carlsbad, CA, USA), and PE-labeled anti-MHC II HLA-DP/DQ/DR (Invitrogen) or a staining control buffer including dye-matched isotype control antibodies (BD and Invitrogen). Cells were incubated at room temperature for 1 h, rinsed with staining buffer, and resuspended in PBS. Samples were read on a FACS Canto II flow cytometer (BD).

### 2.6. Bead-Based Immunoassays

Supernatant cytokine concentrations were measured using bead-based immunoassays for human IL-1β, IL-2, IL-6, IL-8, IL-10, IL-12p70, IFN-γ, RANTES, and MCP-1 (BD) according to manufacturer’s instructions. Bead fluorescence levels were read on a FACSCanto II flow cytometer. Cytokine concentrations were calculated by fitting a 4-parameter logistic curve to cytokine standards included in the kits and applying this formula to sample fluorescence readings.

### 2.7. Statistics

Two-way ANOVA with interaction between treatment group and day of collection was conducted with Statistical Analysis System (SAS: SAS Institute, Cary, NC, USA). Multiple comparison of least square means adjusted with the Tukey approach were performed separately for data from each day. Pairwise Welch’s *t*-tests were also performed between samples. *α* for these *t*-tests was determined with a Bonferroni correction for each experiment.

## 3. Results

### 3.1. Phage K Host Ranges on Military Clinical Isolates

Bacteriophage K purchased from ATCC was isolated as early as 1930 [35]. Before testing its effects on human dendritic cells, we determined the spectrum of phage K lytic activity against a panel of 36 diverse clinical *S. aureus* isolates including 25 MRSA and 11 MSSA strains using the efficiency of plating tests (Table 1). The strains were isolated from military patients with infections of traumatic and surgical wounds, urinary and respiratory tracts and septicemia. Phage K demonstrated a broad host range for both methicillin-resistant and methicillin-susceptible clinical isolates. Overall, 83% (30/36) of strains were susceptible to phage K.

### 3.2. Phage K Stability in PBS

Since phage preparations intended for human cell studies were suspended in PBS (to minimize potential toxic effects on cells), which is a sub-optimal diluent for phage, we tested a short-term phage K stability at 4 °C in PBS including dialyzed and non-dialyzed phage stocks (Table 2). The results showed that the PBS stocks of phage K did not display a considerable reduction in titers at 4 °C at least for one month, while we used the phage preparations for cell culture experiments within two weeks.

### 3.3. Phage K Effects on Human Dendritic Cells

**Figure 1:**
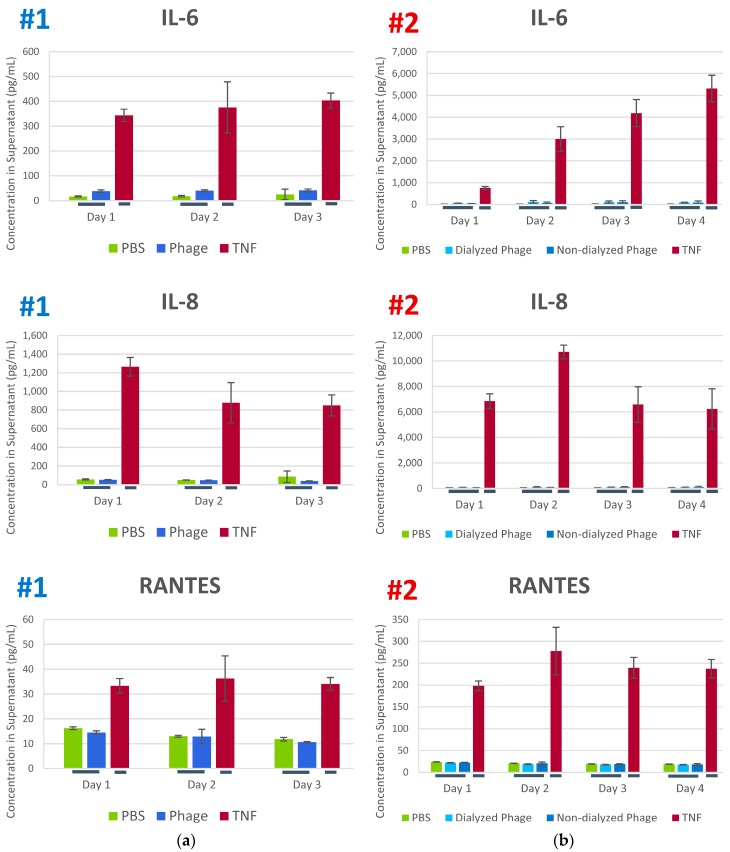
Examples of expression of cytokines IL-6, IL-8 and RANTES in response to phage K, PBS (negative control) and TNF (positive control) in experiments #1 (**a**) and #2 (**b**). Tukey grouping indicated by lines under bar graphs. *p* values can be found in Supplemental Tables.

**Figure 2:**
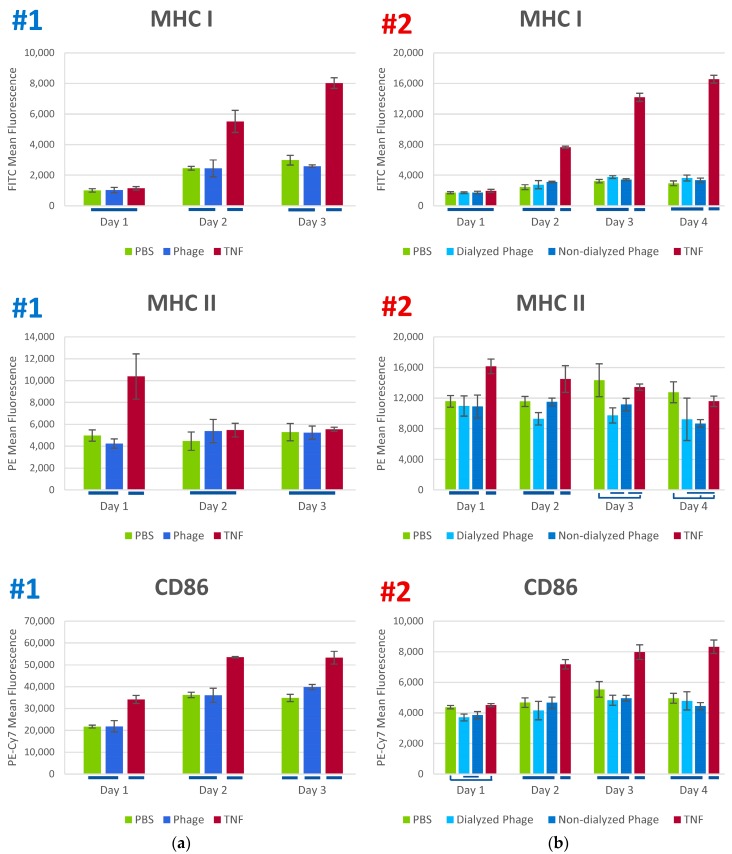
Examples of expression of antigen-presenting and costimulatory surface markers (MHC I, MHC II and CD86) in response to phage K, PBS (negative control) and TNF (positive control) in experiments #1 (**a**) and #2 (**b**). Tukey grouping indicated by lines under bar graphs. *p* values can be found in Supplemental Tables.

We examined the potential inflammatory effects of the staphylococcal phage K in vitro using human monocyte-derived dendritic cells (MoDC). Two iterations of the experiment were conducted with different lots of human monocytes and different batches of phage. Immature MoDC were exposed to either PBS (negative control), phage K in PBS (100 PFU/cell), or TNF (positive activation control). The experiment showed a very limited effect of phage K on expression of a number of cytokines (IL-1β, IL-2, IL-6, IL-8, IL-10, IL-12p70, IFN-γ, MCP-1, and RANTES) and surface markers (MHC I, MHC II, CD80, and CD86). The second experiment differed from the first one by a longer period of observation (four days instead of three) and included a second stock of phage K that had not gone through the extra purification by dialysis step. The results observed in the two experiments (see Figure 1 and Figure 2) were consistent in terms of similar responses of the cells to phage K and the vehicle (PBS).

We observed a potent stimulation of expression of cytokines IL-6, IL-8, and RANTES by TNF-treated samples (positive control) and very weak responses to the K phage and PBS (Figure 1). Several cytokines tested (IL-1β, IL-2, IL-10, IL-12p70, and IFN-γ) showed no measurable response in any experimental group (data not shown). Of the cytokines that showed measurable and consistent responses, there was no statistically significant difference between the PBS control and the phage K treated samples. There was a difference in overall magnitude of cytokine expression observed between the two iterations of the experiment, but this can likely be explained by the use of different lots of monocytes.

In addition to assessing the expression of cytokines, we assessed the maturation of the immature dendritic cells by examining their surface expression of co-stimulatory markers CD80 and CD86, as well as expression of the antigen-presenting markers MHC I and MHC II. In both experiments, expression of MHC I, MHC II, and CD86 was increased by TNF exposure relative to PBS negative controls at one or more timepoints tested, but phage K-treated groups displayed expression profiles similar to the PBS control (Figure 2). CD80 expression could not be assessed from the first experiment due to poor staining, but during the second experiment, it had a similar profile, with expression in the phage K-treated group paralleling the PBS control (Figure S1).

## 4. Discussion

Staphylococcal phage K was isolated around 90 years ago [35] and belongs to the family *Myoviridae*, sub-family *Spounavirinae* and the genus of Twort-like phages [36]. This phage has been shown to lyse from 47% [37] to 84% [38] of *S. aureus* strains and was able to expand its host range after exposure to phage-resistant cultures [39]. Phage K alone [40], in the mix with another phage [41] or phage K modified variants [42] demonstrated significant degradation of biofilms in vitro. In another study, phage K efficiently dispersed *S. aureus* biofilms in central venous catheters in a rabbit model [43]. Phage K also prevented abscess formation in rabbits [44]. Our results on phage K host range for 36 clinical isolates from military patients (83%) were very similar to those of Kelly et al. [38] obtained for 180 *S. aureus* isolates from clinical samples and dairy products (84%).

The results of our cell culture experiments suggest that phage K had little to no effect on the expression of most cytokines and activation markers tested. Similar data that show the lack of cytokine response in vitro in human cells and in vivo in a mouse model have been published for *E. coli* phage T4 and its head proteins [13]. None of three *E. coli* O157:H7 phages changed cytokine levels after oral administration to mice [14].

Our data also suggest that the phage preparations used in this study were sufficiently purified from pathogen-associated molecular patterns of *S. aureus* such as peptidoglycan, lipoteichoic acid and lipoproteins, potent activators of innate immune system that have been shown to enhance the production of cytokines by human dendritic cells [45].

Given that dendritic cells are potent antigen-presenting cells and messengers between the innate and the adaptive immune systems, our reproducible data suggest that staphylococcal phage K likely does not independently initiate an inflammatory reaction or may have a very limited impact. A single phage species may not be representative of other phages, so further investigations should be conducted to establish whether other phages have a different impact. Also, due to natural variations in immune reactions across different patients, this type of study should be conducted with a greater number of donor samples to establish reproducibility across a wider population.

## Figures and Tables

**Table 1:** *S. aureus* strains used in this work.

Strain	Source	Tissue/Organ of Origin	MSSA or MRSA ^1^	Phage K Susceptibility ^2^	Phage K Titer (PFU/mL)	Phage K EOP ^3^
NSCO308	WID ^4^	UNK ^5^	MSSA	S	6.0 × 10^11^	1.00
MRSN18	MRSN ^6^	Wound	MRSA	S	3.0 × 10^11^	0.50
MRSN30	MRSN	Wound	MRSA	S	8.0 × 10^10^	0.13
MRSN42	MRSN	Wound	MRSA	S	2.5 × 10^11^	0.42
MRSN214	MRSN	Wound	MRSA	S	2.5 × 10^10^	0.04
MRSN219	MRSN	Urine	MRSA	S	2.0 × 10^11^	0.33
MRSN250	MRSN	Urine	MRSA	S	1.5 × 10^11^	0.25
MRSN352	MRSN	Wound	MRSA	S	2.5 × 10^11^	0.42
MRSN549	MRSN	Wound	MRSA	S	2.5 × 10^11^	0.42
MRSN563	MRSN	Wound	MRSA	S	2.2 × 10^11^	0.37
MRSN1722	MRSN	Sputum	MRSA	S	1.3 × 10^10^	0.02
MRSN1732	MRSN	Tissue	MRSA	S	4.5 × 10^11^	0.75
MRSN1952	MRSN	Wound	MRSA	S	1.0 × 10^11^	0.17
MRSN2339	MRSN	Wound	MRSA	S	2.5 × 10^10^	0.04
MRSN2763	MRSN	Wound	MRSA	S	9.5 × 10^10^	0.16
MRSN3573	MRSN	Blood	MRSA	R	0	0
MRSN3643	MRSN	Tissue	MRSA	S	1.3 × 10^11^	0.22
MRSN3710	MRSN	Wound	MRSA	S	3.0 × 10^11^	0.50
MRSN3966	MRSN	Blood	MRSA	R	0	0
MRSN4109	MRSN	Sputum	MRSA	S	6.0 × 10^10^	0.10
MRSN4344	MRSN	Wound	MSSA	S	2.0 × 10^11^	0.33
MRSN4531	MRSN	Wound	MRSA	S	4.0 × 10^11^	0.67
MRSN4535	MRSN	Wound	MRSA	S	8.0 × 10^10^	0.13
MRSN5079	MRSN	Wound	MSSA	S	2.0 × 10^11^	0.33
MRSN6168	MRSN	Blood	MSSA	R	0	0
MRSN7983	MRSN	Wound	MSSA	S	1.5 × 10^11^	0.25
MRSN8383	MRSN	Wound	MSSA	S	2.0 × 10^11^	0.33
MRSN9127	MRSN	Sputum	MSSA	S	1.5 × 10^11^	0.25
MRSN9287	MRSN	Wound	MSSA	R	0	0
MRSN9832	MRSN	Nasal swab	MRSA	R	0	0
MRSN9834	MRSN	Nasal swab	MRSA	S	4.0 × 10^10^	0.07
MRSN10110	MRSN	Wound	MSSA	S	1.1 × 10^11^	0.18
MRSN10185	MRSN	Blood	MSSA	S	7.5 × 10^10^	0.12
MRSN12239	MRSN	Eye	MSSA	S	9.5 × 10^11^	1.58
NAJAF22	MRSN	UNK	MRSA	S	1.2 × 10^11^	0.20
NAJAF33	MRSN	UNK	MRSA	R	0	0

^1^ MSSA, methicillin-susceptible *S. aureus*; MRSA, methicillin-resistant *S. aureus.* ^2^ S, susceptible; R, resistant. ^3^ EOP, efficiency of plating. ^4^ Wound Infection Department, Bacterial Diseases Branch, Walter Reed Army Institute of Research. ^5^ UNK, unknown. ^6^ Multidrug-resistant Organism Repository and Surveillance Network, Bacterial Diseases Branch, Walter Reed Army Institute of Research.

**Table 2:** Stability of phage K suspended in PBS.

Phage K Stock	Phage titer (PFU/mL) on:				
	Day 1	Day 9	Day 15	Day 23	Day 31
Non-dialyzed	5 × 10^11^	7 × 10^11^	6 × 10^11^	3 × 10^11^	7 × 10^11^
Dialyzed	7 × 10^11^	5 × 10^11^	6 × 10^11^	4 × 10^11^	3 × 10^11^

## Supplementary Materials

The following are available online at http://www.mdpi.com/1999-4915/10/11/617/s1, Figure S1: CD80 Expression during experiment #2. Supplemental Tables: ANOVA *p* values and Welch’s paired *t*-test *p* values for Experiments 1 and 2.
