# Supplementary figures and images for: Effects of Staphylococcus aureus Bacteriophage K on Expression of Cytokines and Activation Markers by Human Dendritic Cells In Vitro
Source: Viruses. 2018 Nov 8;10(11):617. doi: 10.3390/v10110617 (PMC6266804; doi:10.3390/v10110617)

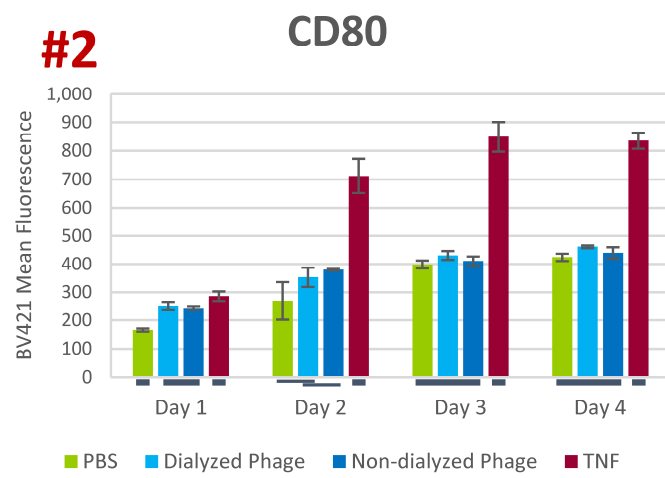

**Supplemental Figure S1.** CD80 expression during experiment #2.

Supplement: Supplementary file 1 [file viruses-10-00617-s001.zip › supplementarys-final/viruses-372358-Figure. S1.pdf]
